# Targeting chromatin binding regulation of constitutively active AR variants to overcome prostate cancer resistance to endocrine-based therapies

**DOI:** 10.1093/nar/gkv262

**Published:** 2015-04-23

**Authors:** Siu Chiu Chan, Luke A. Selth, Yingming Li, Michael D. Nyquist, Lu Miao, James E. Bradner, Ganesh V. Raj, Wayne D. Tilley, Scott M. Dehm

**Affiliations:** 1 Masonic Cancer Center, University of Minnesota, Minneapolis, MN 55905, USA; 2 Dame Roma Mitchell Cancer Research Laboratories and Freemasons Foundation Centre for Mens’ Health, School of Medicine, The University of Adelaide, Adelaide, SA 5005, Australia; 3 Graduate Program in Molecular, Cellular, Developmental Biology and Genetics, University of Minnesota, Minneapolis, MN 55905, USA; 4 Department of Urology, University of Texas Southwestern Medical Center, Dallas, TX 75390, USA; 5 Department of Medical Oncology, Dana-Farber Cancer Institute, Boston, MA 02115, USA; 6 Department of Laboratory Medicine and Pathology, University of Minnesota, Minneapolis, MN 55905, USA

## Abstract

Androgen receptor (AR) variants (AR-Vs) expressed in prostate cancer (PCa) lack the AR ligand binding domain (LBD) and function as constitutively active transcription factors. AR-V expression in patient tissues or circulating tumor cells is associated with resistance to AR-targeting endocrine therapies and poor outcomes. Here, we investigated the mechanisms governing chromatin binding of AR-Vs with the goal of identifying therapeutic vulnerabilities. By chromatin immunoprecipitation and sequencing (ChIP-seq) and complementary biochemical experiments, we show that AR-Vs display a binding preference for the same canonical high-affinity androgen response elements (AREs) that are preferentially engaged by AR, albeit with lower affinity. Dimerization was an absolute requirement for constitutive AR-V DNA binding and transcriptional activation. Treatment with the bromodomain and extraterminal (BET) inhibitor JQ1 resulted in inhibition of AR-V chromatin binding and impaired AR-V driven PCa cell growth *in vitro* and *in vivo*. Importantly, this was associated with a novel JQ1 action of down-regulating AR-V transcript and protein expression. Overall, this study demonstrates that AR-Vs broadly restore AR chromatin binding events that are otherwise suppressed during endocrine therapy, and provides pre-clinical rationale for BET inhibition as a strategy for inhibiting expression and chromatin binding of AR-Vs in PCa.

## INTRODUCTION

The androgen receptor (AR) is a steroid receptor transcription factor that regulates the expression of genes required for development and physiologic function of the prostate gland (1). Additionally, AR transcriptional activity is frequently co-opted by gene fusion events during prostate cancer (PCa) development and progression, as exemplified by AR-dependent ERG overexpression caused by fusion of the ERG gene body to the AR-regulated TMPRSS2 promoter (2). Because of these important developmental and gene fusion signaling roles, inhibiting AR transcriptional activity is a highly effective therapy in patients with advanced PCa. This is achieved by blocking production of androgens and competitive inhibition of the AR ligand binding domain (LBD) with antiandrogens. While these endocrine therapies are initially effective, PCa will eventually progress to a lethal, therapy-resistant disease stage termed castration-resistant PCa (CRPC) (3–5). CRPC is associated with myriad alterations in the androgen/AR axis that promote ongoing AR transcriptional activity. Newer androgen synthesis inhibitors (abiraterone) and antagonists (enzalutamide) were developed to overcome several of the mechanisms underlying persistent AR transcriptional activity, and have been shown to extend overall survival of patients with CRPC (6,7). However, there is evidence that AR transcriptional activity may even resume in patients treated with these second-generation therapies (8). Collectively, these considerations signify a pressing need for more durable AR inhibition strategies in CRPC.

One mechanism proposed to support persistent AR transcriptional activity in CRPC is synthesis of constitutively active AR variant (AR-V) proteins lacking the AR LBD (9–13). Several AR-Vs have been identified and shown to be translated from alternatively-spliced AR mRNAs, which can be highly expressed in CRPC cells harboring structural rearrangements in the AR gene (14–16). AR-Vs have been shown to drive androgen-independent cell proliferation in a manner that is resistant to antiandrogens, including enzalutamide (17). Thus, AR-Vs represent a mechanism whereby the growth of CRPC cells can remain AR-dependent, yet uncoupled from endocrine regulation. Tumors that have developed this mechanism of resistance are unlikely to respond to successive generations of endocrine therapies. Indeed, a recent prospective trial found that mRNA expression of the AR-V7 splice variant in tumor cells circulating in the blood of CRPC patients was associated with primary resistance to abiraterone and enzalutamide (18). These findings indicate that AR-Vs could be attractive targets for therapy of CRPC.

The absence of a LBD presents a formidable challenge for direct inhibition of AR-Vs in CRPC because alternative sites on the AR protein appropriate for specific and high-affinity drug targeting are not evident. An indirect approach may be through inhibition of AR-V transcriptional activation mechanisms. However, there is a paucity of information regarding the molecular activity of AR-Vs in CRPC. To address this limitation, we used chromatin immunoprecipitation followed by massively parallel sequencing (ChIP-seq) and complementary biochemical approaches to understand genome-wide AR-V DNA binding. We find that the mechanisms governing AR-V chromatin interaction resemble those of the prototypical androgen-activated AR: AR-Vs bind DNA as a dimer and utilize canonical inverted repeat androgen response elements (AREs). Moreover, we demonstrate the bromodomain and extraterminal (BET) family inhibitor JQ1 inhibits AR-V mRNA and protein expression, thereby disrupting the AR-V:DNA interaction and repressing the androgen-independent cell growth program driven by AR-Vs.

## MATERIALS AND METHODS

### Cell lines and culture conditions

R1-AD1 and R1-D567 cells have been described (16). LNCaP, 22Rv1, C4-2, 293T and COS-7 cells were obtained from American Type Culture Collection (ATCC). R1AD1, LNCaP, 22Rv1 and C4-2 cells were cultured in Roswell Park Memorial Institute (RPMI) 1640 medium (Invitrogen) with 10% fetal bovine serum (FBS) and antibiotics (100 units/ml penicillin and 100 mg/ml streptomycin). R1-D567 cells were cultured in RPMI 1640 with 10% charcoal-stripped FBS (CSS) and antibiotics. COS-7 cells and 293T cells were cultured in Dulbecco's modified Eagle's medium (DMEM) + 10% FBS and antibiotics. For androgen response experiments, cells were electroporated, seeded in RPMI 1640 containing 10% CSS for 48 h, and stimulated for 24 h with serum free RPMI 1640 medium containing 1 nM mibolerone (Biomol) or dihydrotestosterone (DHT, Sigma), or RPMI 1640 + 10% CSS containing various doses of JQ1. PCa cell lines identities were validated by analysis of signature AR gene alterations (14–16). Cells were cultured in 37°C incubators with 5% CO_2_ for no longer than 15 passages after resuscitation of frozen stocks.

### Antibodies and siRNA reagents

siRNAs targeted to AR exon 1 (siAR1) and exon 3 (siAR2) were purchased from Dharmacon. Antibodies were specific for the AR NTD (Santa Cruz, N-20, #sc-816 or #sc-816X for EMSAs, or Santa Cruz, 441, #sc-7305), ERK-2 (Santa Cruz, D-2, #sc-1647), HA tag (Santa Cruz, F-7, #sc-7392), BRD2 (Cell Signaling, #5848), the TSC2 COOH-terminal domain (Cell Signaling #3612) or AR-V7 (Precision Antibody, #AG10008).

### Plasmids

Plasmid and lentivirus constructs encoding full-length AR, ARv567es, and AR-V7 (AR 1/2/3/CE3) have been described (19). Luciferase reporters containing enhancer elements for FASN AREI, II and III and TSC2 exon 37 ARE were constructed by isolating the desired genomic sites by PCR with forward primers containing XhoI sites and reverse primers containing BglII sites (Supplemental Table S1), digestion with XhoI/BglII, and cloning into a XhoI/BglII-digested pGL4.23 minimal promoter luciferase vector (Promega). Site directed mutagenesis of ARv567es and AR-V7 expression vectors, as well as FASN AREI and TSC2 ARE luciferase reporters, was performed using a site-directed mutagenesis kit (Stratagene) and mutagenic primers listed in Supplemental Table S1.

Control shRNA, shAR#1 or shAR#2 were expressed using the lentiviral vector pLV-MSV40 (19). To generate these shRNA-expressing lentiviral constructs, oligonucleotides (Supplemental Table S1) with the desired shRNA sequences were synthesized such that annealing generated 64-mer cassettes with 5′ BglII and 3′ HindIII-compatible ends for ligation downstream of the histone H1 promoter in the BglII/HindIII-digested pCMS4-H1P-EGFP vector, which has been described previously (20). These H1 promoter/shRNA fragments were then liberated from this vector by digestion with EcoRI/ClaI and ligated with EcoR1/ClaI-digested pLV-MSV40.

The pLV-MSV40-HA vector was derived from pLV-MSV40 by adding a HA tag downstream of SV40 promoter. This was accomplished by PCR of the HA tag fragment from a CMV5 vector with an N-terminal HA tag (21) using a forward primer harboring a Smal site and a reverse primer harboring a SalI site, digestion with SmaI/SalI, and ligation with EcoRV/SalI-digested pLV-VSV40. GFP and AR-V7 fragments were excised from previously-described pLV-MSV40 vectors (19) using EcoRV/SalI and sub-cloned into pLV-MSV40-HA vector digested with EcoRV/SalI.

Construction of transcription activator-like effector nuclease (TALEN) expression vectors targeting the FASN AREI site (Supplemental Table S2) was performed by Golden Gate cloning using the mammalian expression vector pC-GoldyTALEN (Addgene #38143) as described previously (16).

### Multiplex ligation-dependent probe amplification (MLPA)

MLPA assays were performed as described previously (15).

### Western blot

Western blots were performed as described (9). Briefly, blots were incubated with primary antibodies overnight at 4°C and secondary antibodies at room temperature for 1 h. Blots were incubated with Super Signal chemiluminescence reagent (Pierce) and exposed to X-ray film.

### ChIP-seq and ChIP-PCR

Chromatin immunoprecipitation (ChIP) with an antibody specific for the AR NTD (AR-N20, Santa Cruz) was performed for three independent biological replicate experiments as described (22) with the following modifications: R1-AD1 and R1-D567 cells were seeded at 5 × 10^6^ cells/plate on 10 cm plates in RMPI 1640 + 10% CSS, allowed to settle for 72 h, then re-fed for 4h with RMPI 1640 + 5% CSS containing vehicle (ETH) or 1 nM DHT prior to fixation. Nuclear pellets were sonicated on ice for eight cycles at 40% amplitude using a 450 Sonifer (Branson). Each cycle consisted of 10 s pulse/10 s rests for 1 min, with 2 min rests between cycles. Lysates were immunoprecipitated with Protein A/G Plus agarose beads (Santa Cruz Biotechnology) pre-blocked with tRNA (Sigma). DNA was purified using a PCR purification kit (Qiagen). Alternatively, immunoprecipitated complexes were boiled in sodium dodecyl sulphate (SDS) loading buffer and subjected to western blot using a mouse monoclonal antibody specific for the AR NTD (A441, Santa Cruz). For ChIP-seq, 10 ng of DNA (ChIP-enriched or input) was used for library creation with a TruSeq ChIP Sample Preparation Kit (Illumina cat. #IP-202-1012) and sequenced at the U of M Genomics Center using an Illumina HiSeq2000 at 1× 50 bp. Mapping and processing of fastq files were performed in Galaxy (23). Reads were mapped to hg19 with BWA (24) using default parameters. SAMtools was used to remove duplicate and non-uniquely mapping reads (MAPQ cutoff 15) (25). Peaks were called using data from biological replicate 1 with two different software packages, MACS (26) and CisGenome (27), using default parameters, a *P* value cutoff of 1.00E−05 for both packages, and input DNA as negative control. Consensus peaks were determined by including only regions identified by both peak callers; these peaks were merged by using the inner coordinates of the overlapping regions. ChIP-seq data are available through NCBI's Gene Expression Omnibus (GSE61838).

Compressed SRA files from ChIP-seq studies assessing BRD2/3/4 chromatin occupancy in VCaP ((28), GSE55062) and H3K27Ac marks in VCaP ((28), GSE55062) or LNCaP ((29), GSE27823) cells were converted into fastq format using the fastq-dump utility within the SRA toolkit (NCBI). For paired-end sequence data, only read 1 was used for downstream analyses. Mapping and processing of fastq files were performed in Galaxy (23) exactly as described above for analysis of AR ChIP-seq data from R1-AD1 and R1-D567 cells. AR and BRD2/3/4 peak files from VCaP cells (bed format) were a kind gift from Dr Irfan Asangani.

Manipulation of intervals for analyzing overlaps between different ChIP-seq datasets was performed in R 3.0.1 or using Galaxy. Cistrome (30) was used to quantitate conservation of peak sets and generate heatmaps using peak data in .bed format and signal intensity data in wiggle format as input. HOMER (31) and R were used to generate histograms of tag density around peaks. Known androgen response elements (AREs) were identified in peak sets using CisGenome (27) with default parameters. Fold enrichment and significance (Fisher's exact test, calculated using R) of AREs in the experimental peak sets were determined by comparisons to control genomic regions with matched physical distribution. ARE position weight matrices were from the JASPAR CORE vertebrata database (32), or a previous study identifying AREs that are preferentially bound by wild-type AR or an AR/GR hybrid (SPARKI AR, (33), a kind gift provided by Prof. O. Janne). Identification of *de novo* sequence motifs in the peak sets was performed using the Gibbs Motif Sampling approach implemented in CisGenome (parameters altered from defaults: *K* = 10; mean motif length = 15), with enrichment and significance calculated as above. The diffReps tool (34) run in G-test mode with default parameters was used to identify BRD2/3/4 binding sites that were altered in VCaP cells by JQ1 treatment.

Visualization of ChIP-seq data at the gene-track level was performed using Integrated Genomics Viewer (IGV 2.3, Broad Institute, (35)) and tdf files that had been generated from bam files using IGV Tools (default parameters). For manual inspection of AR and ARv567es binding near genes identified in previous studies as being AR-V ‘unique’ (36,37), .bam files from three biological replicates were merged to boost signal intensity.

BRD2 ChIP-PCR was performed exactly as AR ChIP-seq and AR ChIP-PCR, with the exception that cells were fixed with 1.5 mM of freshly prepared ethylene glycolbis(succinimidylsuccinate) (EGS, Thermo Scientific #51565) in 10 ml PBS at room temperature for 30 min. For all ChIP-PCR experiments, DNA (ChIP-enriched or input) was subjected to quantitative PCR using PerfeCTa SYBR Green FastMix (Quanta Biosciences) and gene-specific primers listed in Supplemental Table S1.

### Luciferase reporter gene assays

R1-AD1, R1-D567 and LNCaP cells were electroporated with 200 pmol siRNA or 12μg plasmid DNA as described (16,19). Electroporated cells were re-fed 48 h post-transfection with serum-free medium containing 1 nM mibolerone or DHT or 0.1% ethanol as vehicle control. Cells were harvested after 24h of treatment and Dual Luciferase Assays were performed using a kit (Promega) as per the manufacturer's recommendations. Data were normalized by dividing firefly luciferase activity by *Renilla* luciferase activity,

### Transcription activator-like effector nuclease (TALEN) genome editing

Cells were electroporated with 6μg of left and right TALENs and seeded on 6-well plates in RMPI 1640 + 10% CSS for 96 h. Genomic DNA was extracted using a Nucleospin Tissue Kit (Machery-Nagel #740952). The TALEN-targeted region was amplified by PCR using FASN-specific primers (Supplemental Table S1), gel purified, and cloned for Sanger sequencing or subjected to T7E1 endonuclease assays. T7E1 assays were performed by incubating 400 ng of PCR product in 1× NEBuffer 2 (New England Biolabs) at 98°C for 5 min, slowly cooling to room temperature, digesting with 1 unit of T7 endonuclease 1 (NEB #M0302S) at 37°C for 1 h, and resolving products by electrophoresis in 2% agarose gels.

### Lentivirus transduction

Conditions for lentivirus virus packaging and cell transduction have been described (19).

### Total RNA extraction and quantitative real time RT-PCR

Total RNA was isolated using TRIzol (Invitrogen) and reverse-transcribed (Transcriptor First Strand cDNA Synthesis kit, Roche Applied Science). Quantitative PCR was performed with cDNA using PerfeCTa SYBR Green FastMix (Quanta Biosciences) with primer sets specific for FASN, FKBP5, LIMA1, PSA, hK2 and GAPDH (Supplemental Table S1). Fold changes in mRNA expression levels were calculated using the comparative Ct method as described (19).

### Electrophoretic mobility shift assay (EMSA)

IRD700-labled and competitor duplexes were prepared by annealing complementary synthetic DNA oligonucleotides (Supplemental Table S1). COS-7 cells growing on 10 cm plates were transfected with empty vector or plasmids encoding AR, ARv567es or AR-V7 (AR 1/2/3/CE3) using Lipofectamine 3000 (Invitrogen) according to the manufacturer's recommendations. COS-7 cells transfected with AR were stimulated with 1 nM mibolerone for 1hr prior to nuclear extraction as described (38). Binding reactions were carried out with 5 μg nuclear extract and 2.5 nM of IRD700-labeled FASN AREI duplex using an Odyssey Infrared EMSA Kit (LI-COR # 829-07910) as per the manufacturer's recommendations. For competition assays, unlabeled duplexes were added to binding reactions at 5 or 100 molar excess. For binding saturation experiments, binding reactions were carried out by titrating IRD700-labeled FASN AREI duplexes against a fixed 10 μg of nuclear extract. Binding reactions were resolved in 4% polyacrylamide gels with Tris-glycine electrophoresis buffer (50 mM Tris, 380 mM glycine, 2mM EDTA). Gels in glass plates were scanned in an Odyssey scanner (LI-COR) at the 700 nm channel. Images were captured and specific shift bands were quantified using Image Studio software (LI-COR). Binding saturation plots were developed using GraphPad Prism 5 software, with the apparent equilibrium dissociation constants (*K*_d_) defined as the concentration of IRD700-labeled FASN AREI duplex required to achieve half-maximum binding.

### DNA duplex pull-down assay

Cells were cultured 72 h on 10 cm plates in RMPI 1640 + 10% CSS and stimulated with 1 nM DHT (R1-AD1 cells) or vehicle (ethanol, R1-D567 cells) in RMPI 1640 + 5% CSS for 4 h. Cells were lysed, cleared, and 500 μg of protein lysates were used for DNA duplex pull-downs as described (39) with 1 μg of 5′ biotinylated duplex prepared by annealing complementary synthetic DNA oligonucleotides (Supplemental Table S1). Eluted proteins were analyzed by western blot.

### Gene set enrichment analysis (GSEA)

GSEA was performed using GSEA v2.07 (Broad Institute, (40) using publicly-available datasets (R1-AD1 ± 1 nM DHT = GSE49196, R1-D567 ± siARv567es = GSE49196, LNCaP ± 1 nM DHT = GSE26483). A set of the top 100 genes up-regulated by treatment of LNCaP cells with 10 mM I-BET762, was obtained from a previous study (41). This gene set, termed LNCaP_10uM_UP (Supplemental Table S3) was tested for enrichment in gene expression data from R1-AD1 cells cultured 24 h in 1 nM DHT versus ethanol vehicle control (NCBI gene expression omnibus GEO dataset GSE49169) (16), R1-D567 cells transfected with control siRNA vs. siRNA targeting ARv567es (GEO dataset GSE49169) (16), or LNCaP cultured 18 h in 1 nM DHT versus ethanol vehicle control (GEO dataset GSE26483) using GSEA v2.07 (Broad Institute, (40)). Genes were ranked using the Signal2Noise metric and GSEA was performed against 1000 random gene set permutations.

### Analysis of cell growth by crystal violet assay

Cells were seeded at a density of 5 × 10^4^ cells/well on 24-well plates, allowed to adhere to 48 h, and then treated with JQ1. For experiments combining drug treatment with lentiviral transduction, cells were seeded at 2 × 10^4^ cells/well on 24-well plates, allowed to adhere for 48 h, and then transduced 24 h prior to treatment with JQ1. At the indicated time points, cells were fixed and stained with crystal violet as described (14).

### Mouse xenograft assays

All protocols for mouse experiments were approved by the U of M Institutional Care and Use Committee (IACUC). For enzalutamide treatments, R1-AD1/R1-D567 cell mixtures were prepared by counting R1-AD1 and R1-D567 cells and mixing to achieve final cell number ratios of 90% R1-AD1 and 10% R1-D567. Freshly-prepared admixtures were suspended at 1 × 10^7^cells/ml in 50% RPMI1640 supplemented with 10% FBS and 50% Matrigel (BD Biosciences) and 100 μl was injected subcutaneously in the right flank of male nude mice aged 6–7 weeks (Harlan Laboratories). Tumor size was monitored twice a week by measuring with calipers and calculating tumor volume using the formula length (mm) × width (mm) × height (mm). After tumors reached 100 mm^3^, mice were anesthetized with ketamine/xylazine (100 mg/10 mg/kg), a first tumor biopsy was performed using a 1 mm biopsy punch (Miltex, Inc. York, PA, USA), and then mice underwent surgical castration. Mice were treated with enzalutamide (Selleck Chemicals) by daily oral gavage at 30 mg/kg/day for a total of 7 days. Subsequent tumor biopsies were performed on day 8 (end of enzalutamide therapy) and day 14 (two consecutive tumor measurements displaying increased tumor size). On day 16, the pre-defined study endpoint had been reached (tumor volume ≥ 1000 mm^3^), at which point mice were sacrificed and tumor tissue harvested.

For JQ1 treatments, 100 μl of R1-D567 cell suspension containing 1 × 10^7^ cells/ml in 50% RPMI 1640 supplemented with 10% FBS and 50% Matrigel (BD Biosciences) was injected subcutaneously in the right flank of 14 male nude mice aged 6–7 weeks (Harlan Laboratories). After tumors reached 100 mm^3^, mice were randomized by alternating assignments to control (DMSO, *n* = 7) or treatment (JQ1, *n* = 7) groups, with 250 μl DMSO or 50 mg/kg JQ1 administered by intraperitoneal injection 5 days per week for 4 consecutive weeks. Tumor size was monitored twice a week and mice were euthanized when tumor volume reached 1000 mm^3^, which was the pre-defined ‘survival’ endpoint for this study. Tumor growth rate and survival analyses were performed using GraphPad Prism 5.0 software (La Jolla, CA, USA).

## RESULTS

### Enzalutamide resistance in a treatment model of heterogeneous CRPC

**Figure 1.**
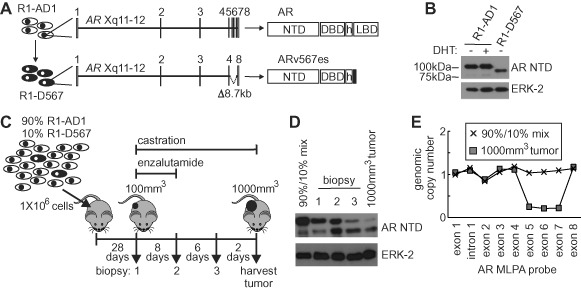
Outgrowth of AR-V expressing cells in a treatment model of heterogeneous CRPC. (**A**) Schematic representation of isogenic prostate cancer cell lines R1-AD1 and R1-D567 expressing AR and ARv567es, respectively. (**B**) Western blots with R1-AD1 and R1-D567 lysates using antibodies specific for the AR NTD and ERK-2 (loading control). (**C**) Schematic of treatment enrichment experiment. Xenografts were established in intact male mice from a 90%/10% admixture of R1-AD1/R1-D567 cell lines. When tumors reached 100 mm^3^, mice were castrated and initiated 7-day treatment with 30 mg/kg/day enzalutamide by oral gavage. Biopsies of xenografts were collected at indicated days and the mice were euthanized when tumors reached 1000 mm^3^. (**D**) Western blot analysis of protein lysates from tumor tissue collected as in (C) probed with antibodies specific for the AR NTD and ERK-2 (loading control). (**E**) Multiplex ligation-dependent probe amplification (MLPA) with genomic DNA isolated from pre- and post-implantation samples. Plots illustrate genomic copy number at indicated genomic locations across the AR gene.

AR-V-expressing tumor cells are common features of the subclonal architecture of heterogeneous CRPC cell lines and tissues, and have emerged as an important component of resistance to endocrine therapies (10–12,15–18,42–45). R1-D567 is a cell line harboring an engineered genomic deletion of AR exons 5–7, which was developed to model an AR gene rearrangement discovered in heterogeneous CRPC tissue (15,16). Whereas the parental R1-AD1 cell line expresses full-length AR, R1-D567 cells display exclusive expression of the constitutively active AR-V, ARv567es (Figure 1A and B). To test the impact of AR-V-expressing tumor cells on the therapeutic response of heterogeneous CRPC, we developed subcutaneous xenografts with a 90%/10% mixture of R1-AD1/R1-D567 cells and treated mice with a combination of castration and enzalutamide (Figure 1C).

Western blot analysis of biopsied tumor tissue that had been established in intact mice revealed that full-length AR remained the predominant protein species (Figure 1C and D). However, serially-sampled tumor tissue revealed enrichment of ARv567es expression relative to full-length AR 6 days after initiation of treatment with enrichment continuing through the study endpoint. DNA copy number analysis via multiplex ligation-dependent probe amplification assay revealed that enrichment of ARv567es expression was due to outgrowth of R1-D567 cells (Figure 1E). This *in vivo* model highlights the importance of understanding the function and regulation of AR-Vs in CRPC, as this may reveal opportunities for novel therapeutic interventions that inhibit AR-V activity and overcome therapeutic resistance.

### ARv567es displays preferential genome-wide binding to canonical AREs

**Figure 2.**
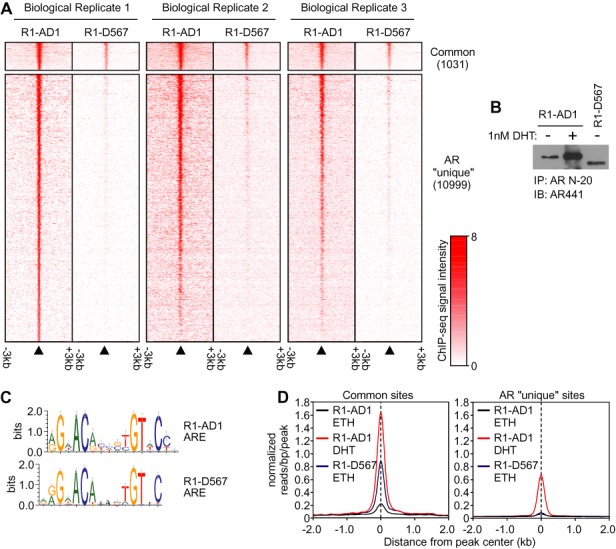
Genome-wide binding of ARv567es to canonical AREs. (**A**) Heatmap of ChIP-seq signals ± 3 kb around R1-AD1 AR peak midpoints from three biological replicate experiments for a set of binding sites identified by peak calling with biological replicate 1 data as being common to dihydrotestosterone (DHT)-treated R1-AD1 and vehicle (ethanol, ETH)-treated R1-D567 cells (upper panel), or ‘unique’ to R1-AD1 cells (lower panel). (**B**) Western blot of chromatin processed for ChIP as in (A) probed with a monoclonal antibody specific for the AR NTD (AR441). (**C**) Sequence motifs enriched at AR (R1-AD1) and ARv567es (R1-D567) binding sites identified *de novo* using the Gibbs Motif Sampling approach. (**D**) Average ChIP-seq tag intensities expressed in mapped reads per base pair per peak normalized per 10^6^ reads from three datasets at binding sites identified as common to R1-AD1 and R1-D567 cells, or ‘unique’ to R1-AD1 cells.

We used the R1-AD1/R1-D567 model to study genome-wide AR and ARv567es binding via ChIP-seq using an antibody targeted to the AR NH_2_-terminal domain (NTD). Peak calling with data from a single ChIP-seq experiment resulted in the identification of 12 030 AR binding sites in R1-AD1 cells and 3554 ARv567es binding sites in R1-D567 cells (Figure 2A and B, Supplemental Table S4). Motif analysis revealed enrichment of canonical androgen response elements (AREs) in both R1-AD1 and R1-D567 cells (Figure 2C, Supplemental Table S5). Further motif analysis revealed that occurrences of stringent and relaxed specificity AREs (33) were similar in both datasets (Supplemental Table S6). These data indicate that regulation of AR and ARv567es DNA binding may proceed through similar mechanisms.

**Figure 3.**
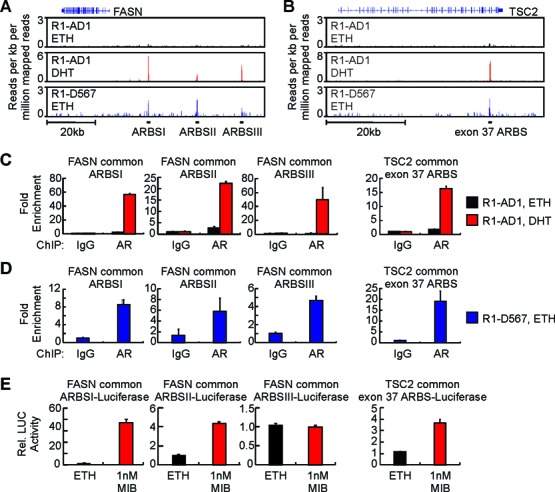
Common AR/ARv567es binding sites function as androgen-responsive enhancers. (**A**) Gene track view of ChIP-seq data at the FASN locus. Common AR/ARv567es binding sites (ARBS) are indicated. Data are from cells treated with dihydrotestosterone (DHT) or vehicle (ethanol, ETH) as indicated. (**B**) Gene track view of ChIP-seq data at the TSC2 locus. A common AR/ARv567es binding site in TSC2 exon 37 is indicated. (**C**) Validation of androgen-mediated recruitment of AR to FASN and TSC2 ARBSs in R1-AD1 cells by ChIP-qPCR. (**D**) Validation of constitutive ARv567es binding to FASN and TSC2 ARBSs in R1-D567 cells by ChIP-qPCR. (**E**) FASN and TSC2 ARBSs were tested for enhancer response to the synthetic androgen mibolerone (MIB) by luciferase reporter assay in R1-AD1 cells.

Integration of R1-AD1 and R1-D567 ChIP-seq datasets identified 1031 common genomic sites engaged by AR and ARv567es (Figure 2A). These sites represented the highest-affinity binding sites for both AR and ARv567es, although the signal for ARv567es occupancy at these sites was lower than for AR (Figure 2D). Occupancy of both factors at these high-affinity sites was apparent in two additional independent biological replicate ChIP-seq experiments (Figure 2A). Examples of these high-affinity binding sites, which were confirmed by ChIP-PCR, mapped to the FASN upstream regulatory region and exon 37 of the TSC2 gene (Figure 3A–D). This approach also identified a set of 10 999 AR ‘unique’ binding sites, representing peaks called in R1-AD1 but not R1-D567 cells (Figure 2A and D). However, ChIP-seq heatmap plots of data from three independent biological replicate experiments provided evidence for weak ARv567es occupancy at many of these sites (Figure 2A), suggesting that at least some were false negatives (i.e. below the threshold of detection of peak calling software). Indeed, directed ChIP-PCR analysis of several of these AR ‘unique’ sites confirmed binding of ARv567es (Supplemental Figure S1).

Integrative analysis of ChIP-seq data also identified a putative set of 2523 ARv567es ‘unique’ binding sites in R1-D567 cells (Supplemental Figure S2A). Inspection of a subset of these sites where enrichment was highest for ARv567es-specific signal revealed that most were located in repetitive genomic elements, suggesting that at least some were false positives (Supplemental Table S7). In line with this, ARv567es-specific occupancy at these sites was not observed in two independent biological replicate ChIP-seq experiments (Supplemental Figure S2A). Further inspection of ChIP-seq data at the gene-track level and directed ChIP-PCR at representative ARv567es ‘unique’ sites that were not within repetitive elements could not confirm ARv567es binding (Supplemental Figure S3). Additionally, the putative ARv567es ‘unique’ sites lacked sequence conservation, unlike common or AR ‘unique’ sites (Supplemental Figure S2B). In aggregate, these findings strongly support the concept that the genome-wide binding preference of constitutively active ARv567es closely resembles that of androgen-activated AR.

The finding of similar AR and ARv567es genomic binding preferences is not in agreement with previous studies reporting AR-V ‘unique’ transcriptional functions and/or genomic binding specificity (11,37,46). To address this directly, we consolidated all mapped ChIP-seq reads from the three biological replicates to boost signal intensity (yielding over 80 × 10^6^ mapped reads per condition), and used these consolidated datasets to manually search for an ARv567es-specific signal at 27 genes reported to be unique targets of AR-Vs but not AR (36,37) (Supplemental Table S8). At many of these candidate loci, recruitment of both AR and ARv567es was apparent (for example, UBE2C and EDN2, Supplemental Figure S4, A and B), while at other candidate loci recruitment of AR but not ARv567es was observed (for example, ZWINT, Supplemental Figure S4C). However, across this entire set of reported AR-V ‘unique’ targets, in no case was an ARv567es-unique signal observed within ±100 kb of the candidate gene (Supplemental Table S8).

### ARv567es activates canonical AREs

**Figure 4.**
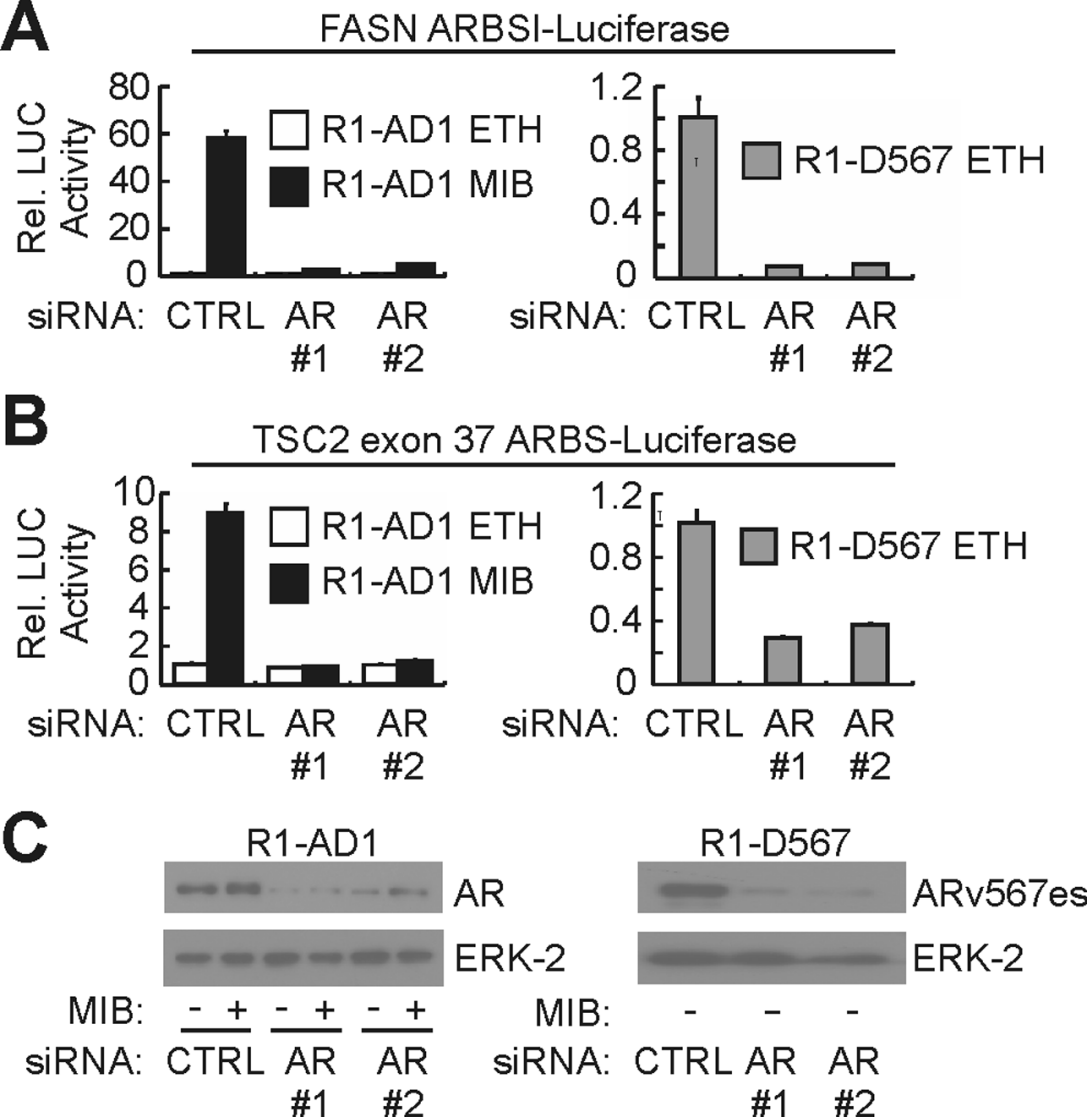
Common AR/ARv567es binding sites are AR/ARv567es-dependent enhancers. (**A**) AR and ARv567es responsiveness of FASN ARBSI was tested by luciferase reporter assays in R1-AD1 and R1-D567es cells transfected with control (CTRL) siRNA or two separate siRNAs targeting AR. Cells were treated with 1 nM mibolerone (MIB) or ethanol (ETH) as vehicle control as indicated. (**B**) AR and ARv567es responsiveness of TSC2 exon 37 ARBS was assessed as in (A). (**C**) Western blots with R1-AD1 and R1-D567 lysates transfected as in (A) and (B) using antibodies specific for the AR NTD and ERK-2 (loading control).

Because our data indicated that ARv567es displays a genome-wide binding preference for canonical AREs, we sought to understand the details of this binding mechanism. First, we tested the ability of common AR/ARv567es binding sites to enhance activity of a luciferase reporter regulated by a minimal core promoter (Figure 3E). Of the three discrete binding sites in the FASN upstream regulatory region, the site most proximal to the FASN transcription start site, termed ARBSI, displayed robust androgen-responsive enhancer activity in luciferase reporter assays (Figure 3E). Knock-down of AR abolished androgen-mediated induction of the FASN-ARBSI reporter, as well as the reporter regulated by the common AR/ARv567es binding site identified in exon 37 of the TSC2 gene (Figure 4A–C). Similarly, knock-down of ARv567es in R1-D567 cells inhibited androgen-independent activity of these reporters (Figure 4A–C).

**Figure 5.**
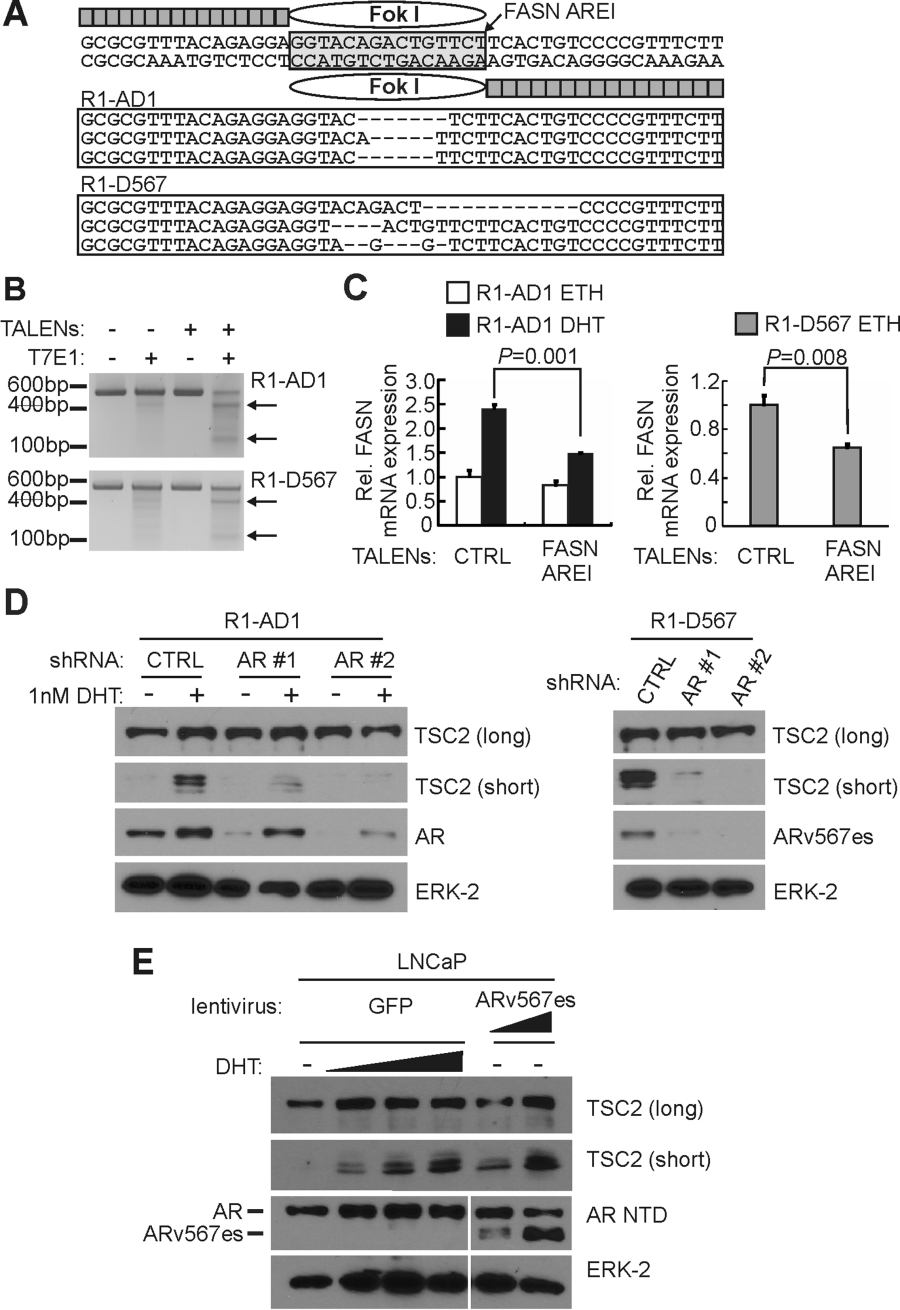
Common AR/ARv567es binding sites regulate endogenous transcriptional outcomes. (**A**) Schematic of TALENs targeted to an androgen response element (ARE) in FASN ARBSI and examples of mutations recovered by PCR from TALEN-transfected R1-AD1 and R1-D567 cells. (**B**) T7E1 endonuclease assays evaluating frequency of mutations (assessed by T7E1 cleavage efficiency) in TALEN-transfected R1-AD1 and R1-D567 cells. (**C**) Quantitative RT-PCR analysis of FASN mRNA levels in R1-AD1 and R1-D567 cells transfected with TALENs targeting FASN AREI or control (CTRL) TALENs targeting an alternate genomic site. Cells were treated with dihydrotestosterone (DHT) or ethanol (ETH) as vehicle control as indicated. (**D**) Western blots were performed with lysates of R1-AD1 (left) and R1-D567 (right) cells transfected with control (CTRL) siRNA or two separate siRNAs targeting AR and treated with DHT or ETH as vehicle control as indicated. Blots were probed with antibodies specific for the COOH-terminal domain of TSC2, the AR NTD or ERK-2 (loading control). Long and short forms of TSC2 are indicated. (**E**) Western blots were performed with lysates of LNCaP cells transduced with lentivirus encoding GFP (control) or ARv567es and treated with a range of DHT concentrations (0.1, 1.0, 10 nM) or ethanol (‘–’, vehicle control) as indicated. Blots were probed as in (D).

Consistent with responsiveness to AR and ARv567es, a consensus inverted repeat ARE, termed FASN-AREI, was identified within FASN ARBSI (Figure 5A). To test the functional importance of FASN-AREI within the context of the endogenous gene locus, we designed transcription activator-like effector nucleases (TALENs) to genomic sequences flanking FASN-AREI, which would result in targeted DNA double-strand (dsDNA) breaks within the ARE core sequence (Figure 5A). In R1-AD1 and R1-D567 cells transfected with these TALENs, PCR of the target site yielded efficient recovery of sequences displaying FASN-AREI mutations caused by imprecise dsDNA break repair (Figure 5A). Further analysis of these PCR products using T7E1 nuclease heteroduplex assays indicated that TALEN-mediated mutations occurred in approximately half of the endogenous FASN AREI sites (Figure 5B). Importantly, these FASN AREI mutations reduced androgen-mediated induction of FASN expression in R1-AD1 cells, as well as constitutive androgen-independent FASN expression in R1-D567 cells (Figure 5C).

A TALEN mutagenesis strategy was not pursued for an ARE located in the TSC2 exon 37 ARBS, as it would lead to mutations in TSC2 coding sequence. However, AR or ARv567es knock-down in R1-AD1 or R1-D567 cells resulted in decreased expression of the TSC2 short isoform arising from transcription initiation within an alternative internal promoter near this ARE site (Figure 5D), which has been shown in a previous study to enhance LNCaP proliferation (47). Additionally, treatment of androgen dependent LNCaP cells with androgens or transduction with lentivirus encoding ARv567es increased expression of the TSC2 short isoform (Figure 5E).

### AR-Vs bind and activate AREs through a dimerization-dependent mechanism

**Figure 6.**
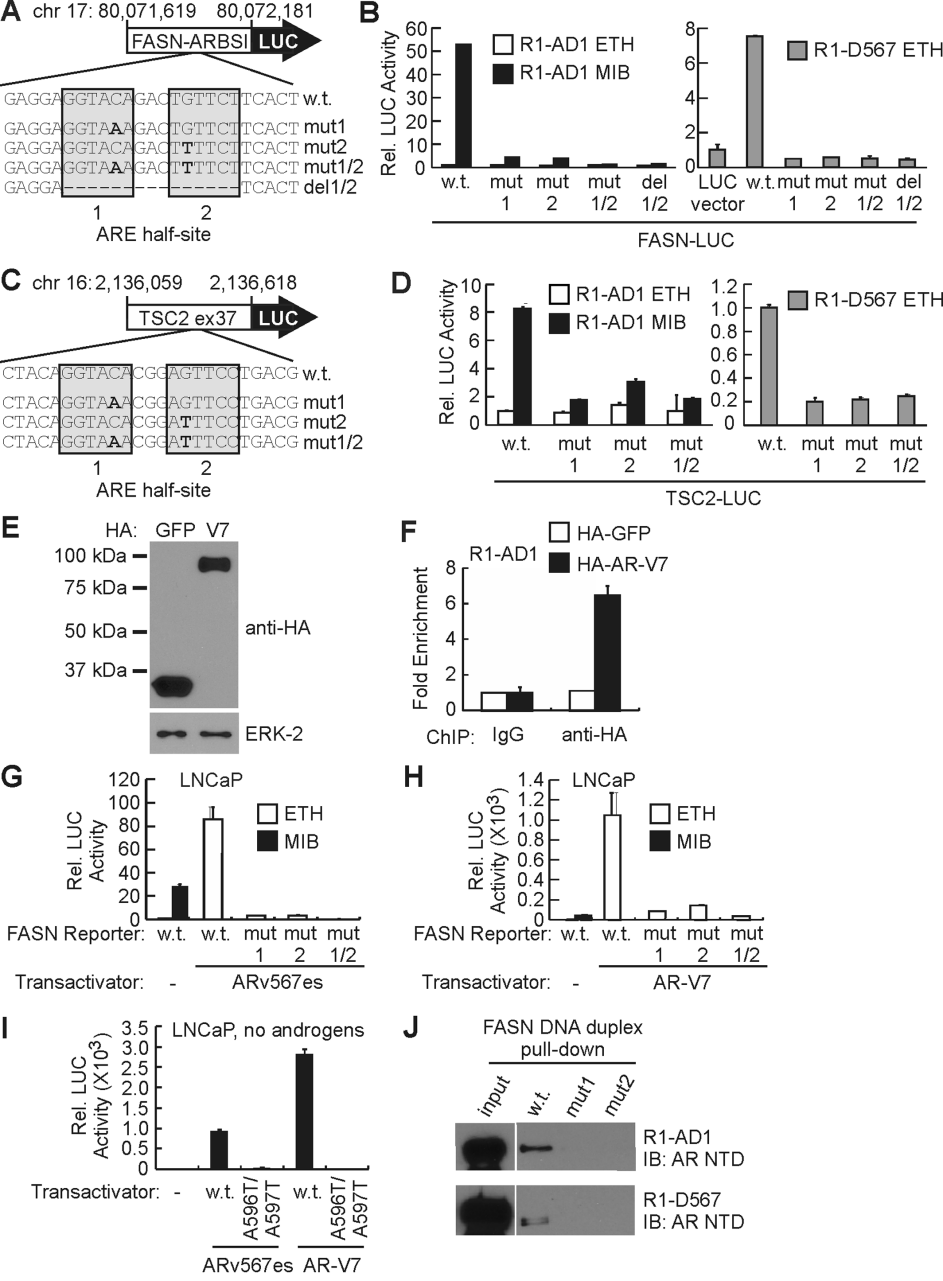
ARv567es binds canonical AREs through a dimerization-dependent mechanism. (**A**) ARE point mutations introduced in FASN ARBSI-LUC. (**B**) Activities of constructs illustrated in (A) were tested in R1-AD1 and R1-D567 cells by luciferase assay. Cells were treated with 1 nM mibolerone (MIB) or ethanol (ETH) as vehicle control as indicated. (**C**) ARE point mutations introduced in TSC2 exon 37-LUC. (**D**) Activities of constructs illustrated in (C) were evaluated by luciferase assay as in (B). (**E**) Western blot of lysates from R1-AD1 cells transfected with HA-GFP and HA-AR-V7 for the ChIP experiment shown in (F). (**F**) Constitutive recruitment of HA-tagged AR-V7 to the FASN ARBS1 site in transfected R1-AD1 cells was tested by ChIP-PCR. Data represent fold enrichment of PCR signal in ChIP DNA isolated using an HA-directed antibody versus non-specific IgG control (which was arbitrarily set to 1). (**G**) Activities of constructs illustrated in (A) were tested by luciferase assay using LNCaP cells transfected with an ARv567es expression vector and treated with 1 nM mibolerone (MIB) or ethanol (ETH, vehicle) as indicated. (**H**) Activities of constructs illustrated in (A) were tested by luciferase assay using LNCaP cells transfected with an AR-V7 expression vector exactly as described in (G). (**I**) Transcriptional activities of wild-type and A596T/S597T D-box mutant versions of ARv567es and AR-V7 were tested in LNCaP cells by luciferase assay as described in (G). (**J**) DNA duplex pull-down assays were performed by incubating biotinylated FASN AREI DNA duplexes harboring core sequences shown in A with cellular extracts from R1-AD1 and R1-D567 cells.

Both the FASN AREI and the TSC2 exon 37 ARE are canonical inverted repeats representative of AREs enriched at genome-wide AR and ARv567es binding sites (Figure 2B). Point mutations disrupting either half site in these AREs abolished androgen-mediated induction by AR in R1-AD1 cells as well as androgen-independent induction by ARv567es in R1-D567 cells (Figure 6A–D). These data indicate that ARv567es binds genomic DNA through a dimerization-dependent mechanism. To test whether this was a general property of AR-Vs, we performed a similar set of assays with AR-V7, which harbors a different COOH-terminal sequence compared with ARv567es, most notably lacking the AR hinge region (19). First, we confirmed binding of HA-tagged AR-V7 to FASN ARBS1 by ChIP-PCR (Figure 6E and F). Similar to ARv567es, point mutations disrupting either half site of FASN ARE1 abolished androgen-independent induction by AR-V7 in LNCaP cells (Figure 6G and H).

To further test the functional requirement for AR-V dimerization, we generated A596T/S597T compound mutations, which have been shown to disrupt the AR D-box homodimer interface (48). Similar to ARE half-site mutations, these mutations abolished transcriptional activity of ARv567es and AR-V7 in LNCaP cells (Figure 6I). These data indicate that dimerization is a general functional requirement for AR-Vs. In agreement with this notion, AR from R1-AD1 lysates and ARv567es from R1-D567 lysates bound to a biotinylated DNA duplex with both FASN AREI half-sites intact, but not to biotinylated DNA duplexes with mutations disrupting either half site (Figure 6J).

**Figure 7.**
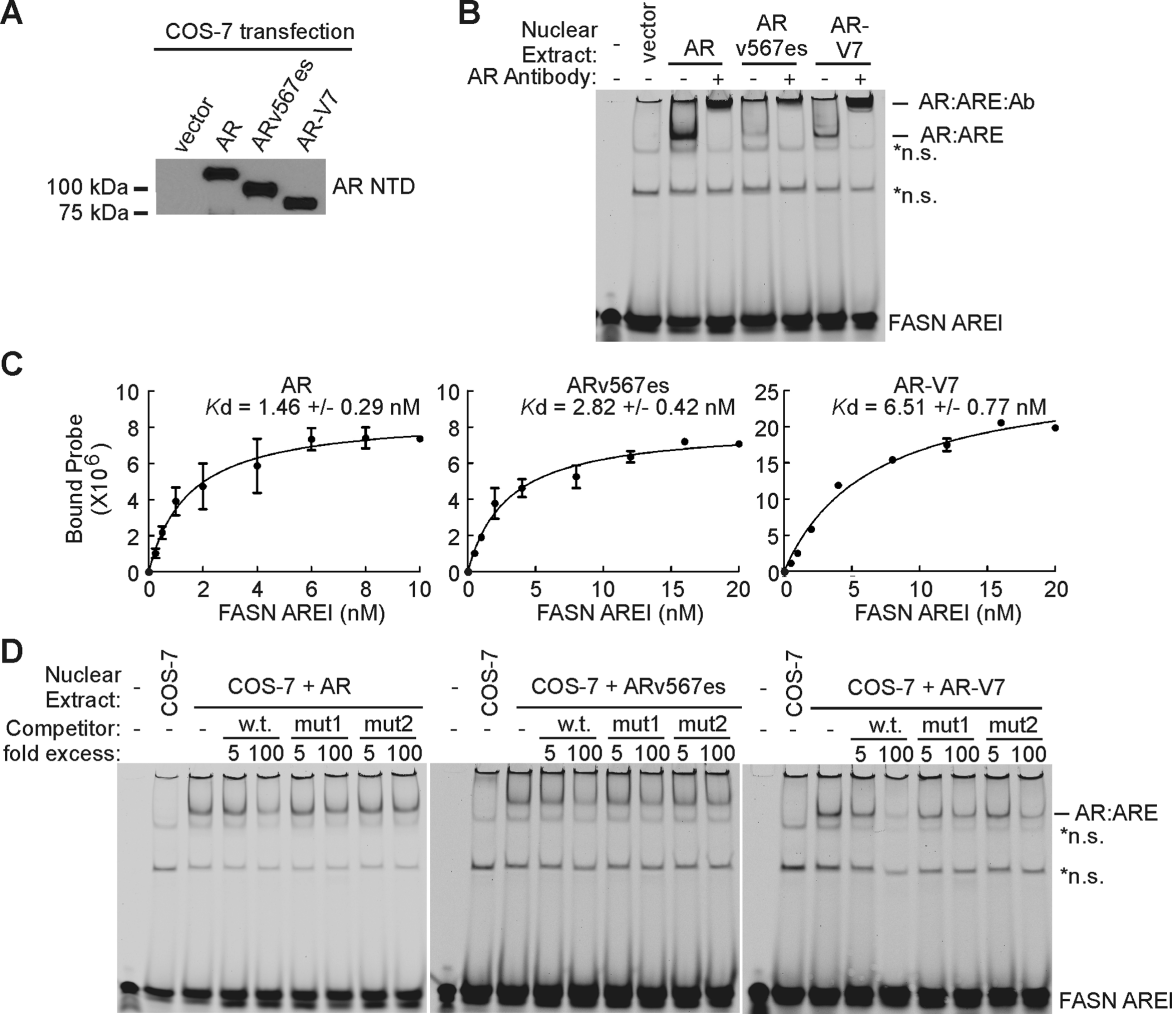
AR-Vs bind a canonical ARE with lower affinity than full-length AR. (**A**) Representative western blot of lysates from COS-7 cells transfected with plasmids encoding AR, ARv567es and AR-V7 for EMSA experiments. The blot was probed with an antibody specific for the AR NTD. (**B**) Binding of AR, ARv567es, and AR-V7 to an IRD700-labeled FASN AREI DNA duplex was assessed by electrophoretic mobility shift assay (EMSA). Supershifts were achieved by adding an antibody specific for the AR NTD to binding reactions as indicated. (**C**) EMSAs were performed as in (B). Labeled FASN AREI duplexes were titrated against a fixed 10 μg of nuclear extract. Apparent equilibrium dissociation constants (*K*_d_) are defined as the concentration of FASN AREI duplex required to achieve half-maximum binding. (**D**) EMSAs were performed as in (B). Unlabeled competitor DNA duplexes harboring wild-type or mutant FASN ARE1 core sequences shown in Figure 6A were added at 5× and 100× molar excess as indicated.

We next tested binding to the FASN AREI site by electrophoretic mobility shift assays (EMSAs) with lysates from COS-7 cells expressing ectopic AR and AR-Vs (Figure 7A). Interestingly, this approach revealed that ARv567es and AR-V7 bound to the FASN AREI site with respective equilibrium dissociation constants (*K*_d_) 2- and 4-fold lower than full-length AR (Figure 7B and C). This lower affinity binding is consistent with the generally weaker signal observed in ChIP-seq experiments for genome-wide ARv567es binding compared with full-length AR binding (Figure 2A and D). For all AR species, FASN AREI binding was susceptible to competition with an unlabeled FASN AREI sequence, but not FASN AREI sequences harboring mutations in either half site (Figure 7D). From these data, we conclude the predominant mode of AR-V binding to genomic DNA is through dimerization-dependent engagement with the same canonical AREs engaged by full-length AR. However, despite this being the predominant mode of genome-wide binding, AR-Vs engage these canonical AREs with a lower affinity than full-length AR.

### Down-regulation of AR and AR-V expression by BET inhibition

**Figure 8.**
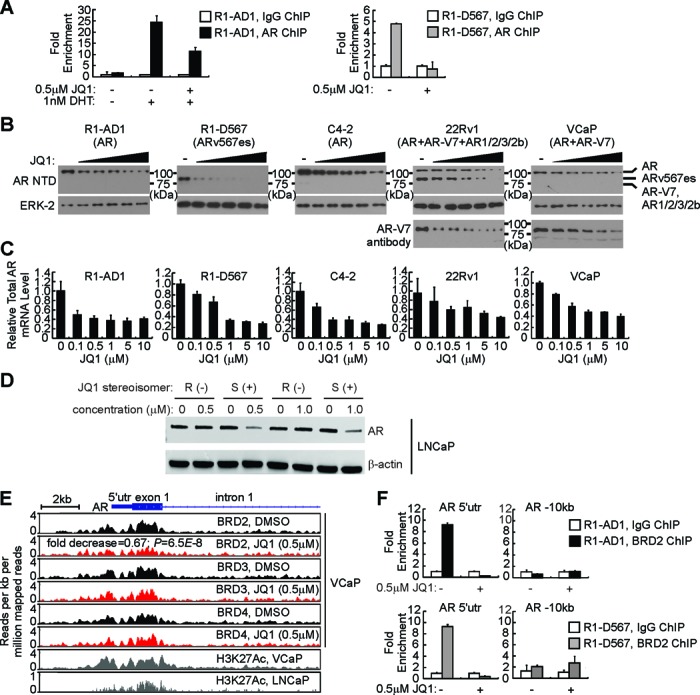
BET inhibitors repress expression of AR and ARv567es in prostate cancer cells. (**A**) Binding of full-length AR or ARv567es to FASN ARBS1 was tested in R1-AD1 or R1-D567 cells, respectively, treated with vehicle (DMSO) or JQ1 (0.5 mM) in the presence or absence of 1 nM dihydrotestosterone (DHT) as indicated. (**B**) Western blots with antibodies specific for the AR NTD, AR-V7 or ERK-2 (loading control) with lysates from indicated PCa cell lines treated 24 h with vehicle (DMSO) or JQ1 (doses: 0.1, 0.5, 1, 5, 10 μM) in medium containing 10% CSS. (**C**) Quantitative RT-PCR analysis of total AR mRNA levels in PCa cell lines treated as in (B). (**D**) Western blots with antibodies specific for the AR NTD or β-actin (loading control) with lysates from LNCaP cells treated 24 h with vehicle (DMSO) or active S(+) or inactive R(–) JQ1 stereoisomers as indicated. (**E**) Gene track view of BRD2, BRD3, BRD4 and H3K27Ac ChIP-seq data at the AR locus. Data were obtained from NCBI Gene Expression Omnibus (GEO), representing VCaP cells treated with 0.5 μM JQ1 or vehicle control (DMSO) (GSE27823, (28)) or DHT-treated LNCaP cells (GSE27823, (29)). The FDR-adjusted *P* value signifying JQ1-mediated loss of BRD2 binding at this site in VCaP cells was derived using diffReps (34). (**F**) Binding of BRD2 to the AR 5′utr region was tested in R1-AD1 or R1-D567 cells treated with vehicle (DMSO) or JQ1 (0.5 μM) as indicated.

Based on these mechanistic findings, we hypothesized that disruption of AR-V:ARE interactions may represent a viable strategy for overcoming AR-V-mediated therapeutic resistance in CRPC. To develop preclinical support for this chromatin targeting concept, we evaluated JQ1, an inhibitor of the BET family of chromatin readers (49) shown to impair genome-wide chromatin binding and transcriptional activation of androgen-activated full-length AR (28). BET family chromatin readers BRD2, 3 and 4 engage with acetylated lysine residues, including H3K27Ac, which constitute chromatin marks enriched at active enhancers and associated transcription start-sites of transcriptionally active genes (50). As expected, JQ1 reduced androgen-mediated AR engagement with FASN ARBSI in R1-AD1 cells (Figure 8A). More strikingly, JQ1 completely inhibited constitutive AR-V engagement with this site in R1-D567 cells (Figure 8A). Unexpectedly, we noted loss of AR and ARv567es protein when R1-AD1 and R1-D567 cells were treated with therapeutically-relevant doses of JQ1 (28,49–51), which corresponded with down-regulated AR mRNA expression (Figure 8B and C). The CRPC C4-2, 22Rv1 and VCaP cell lines also displayed dose-dependent reductions in AR and/or AR-V expression in response to JQ1 treatment (Figure 8B and C). In the LNCaP cell line, down-regulated AR expression was observed for (+)-JQ1 but not (−)-JQ1, indicating specificity for the active stereoisomer (Figure 8D) (49).

To understand the basis for JQ1-mediated down-regulation of AR expression, we analyzed publicly-available ChIP-seq data from studies assessing genome-wide H3K27Ac marks and the effects of JQ1 on BRD2, 3 and 4 binding in LNCaP and VCaP cells (28,29). Interestingly, enrichment of H3K27Ac was evident near the transcription start site of the AR gene, encompassing the 5′ untranslated region (utr) and exon 1 (Figure 8E). Moreover, binding of BRD2, 3 and 4 was apparent in this same region in VCaP cells, with JQ1 treatment resulting in reduced signal intensity for binding of BRD2, but not BRD3 or 4 (Figure 8E). Consistent with this finding, directed ChIP-PCR analysis confirmed robust JQ1-mediated inhibition of BRD2 binding to the AR 5′utr in R1-AD1 and R1-D567 cells (Figure 8F).

### BET inhibitors impair AR and AR-V transcriptional activation and repression

**Figure 9.**
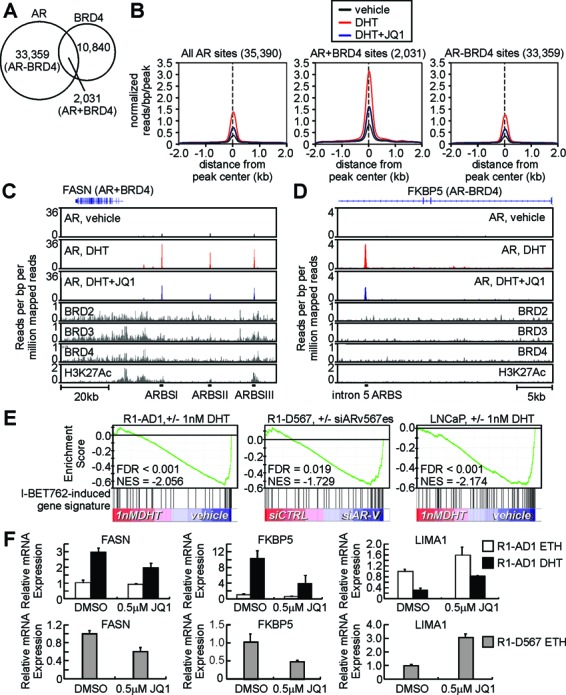
BET inhibitors coordinately suppress transcriptional activation and repression of AR and ARv567es target genes by reducing overall AR chromatin occupancy. (**A**) Venn diagram representing AR and BRD4 chromatin occupancy in VCaP cells. Occupancy data were obtained from a previous study (28). (**B**) Average ChIP-seq tag intensities expressed in mapped reads per base pair per peak normalized per 10^6^ reads from three datasets (VCaP cells treated with vehicle, DHT, or DHT and JQ1) obtained from NCBI GEO (GSE27823, (28)). (**C**) Gene track views of AR, BRD2, BRD3, BRD4 and H3K27Ac ChIP-seq data obtained from NCBI GEO (GSE27823, (28)) at the FASN locus. AR binding sites (ARBS) classified as co-occupied by AR and BRD4 (AR+BRD4) are indicated. (**D**) Gene track views of the FKBP5 locus developed as in (C). An ARBS classified as occupied by AR but not BRD4 (AR-BRD4) is indicated. (**E**) Gene set enrichment analysis demonstrating that AR transcriptional activity in R1-AD1 and LNCaP cells, and ARv567es transcriptional activity in R1-D567 cells, is negatively enriched for a set of I-BET762-induced genes. (**F**) Quantitative RT-PCR analysis of FKBP5, FASN and LIMA1 mRNA expression in R1-AD1 and R1-D567 cells treated with combinations of vehicle (DMSO and ethanol, ETH), 0.5 μM JQ1 or 1 nM DHT as indicated for 24 h.

In a previous study, the inhibitory effects of JQ1 on AR chromatin binding were attributed to a direct interaction between AR and BRD4 (28). We hypothesized our novel finding of down-regulated AR and AR-V expression following JQ1 treatment represented an important component of the JQ1 anti-AR mechanism of action, reminiscent of down-regulated MYC expression in multiple myeloma cells (50,51). To test this, we compared the effect of JQ1 on AR chromatin binding in VCaP cells at AR binding sites co-occupied by BRD4 (denoted AR+BRD4 sites), or AR binding sites not occupied by BRD4 (denoted AR-BRD4 sites, Figure 9A). Whereas AR+BRD4 sites appeared to be higher-affinity AR binding sites than AR-BRD4 sites, JQ1 reduced AR binding by approximately 50% at both of these sub-classes of AR binding sites (Figure 9B). Inspection of specific AR+BRD4 and AR-BRD4 sites (which included FASN-ARBSI and an AR binding site in intron 5 of FKBP5, respectively) confirmed that the presence of H3K27Ac or BET family proteins was not required for JQ1-mediated inhibition of AR binding (Figure 9C and D).

Next, we considered the possibility that the observed reduction in AR mRNA and protein levels could be a non-selective result of the progressive, global decline in cellular mRNA expression that has been shown to ensue following BET inhibition (50). To test this, we focused on a subset of genes that displayed paradoxical induction by BET inhibitor treatment in AR-expressing PCa cells (Supplemental Table S3) (41), hypothesizing these may represent androgen/AR-repressed target genes. Because BET family proteins are associated with transcriptionally active chromatin, but not transcriptionally repressed chromatin (50), we postulated that induction (or de-repression) of these putative AR or AR-V repression targets by BET inhibitors would signify functional selectivity of down-regulated AR expression. Indeed, analysis of gene expression microarray datasets using gene set enrichment analysis (40) demonstrated that transcriptional programs of active AR or AR-Vs were negatively enriched for a set of genes that were induced in LNCaP cells upon treatment with the JQ1-related BET inhibitor I-BET762 (Figure 9E) (41). This was further supported by quantitative RT-PCR, which demonstrated that JQ1 impaired the ability of AR and ARv567es to repress expression of LIMA1 as well as activate expression of FKBP5 and FASN (Figure 9F). Collectively, these results indicate that JQ1 has selectivity in down-regulating AR and AR-V expression, which leads to reduced genome-wide chromatin occupancy and coordinate inhibition of these factors’ transcriptional activation and repression activities. Therefore, inhibition of AR and AR-V expression represents a previously-unknown, yet functionally important, activity of BET inhibitors in PCa cells.

**Figure 10.**
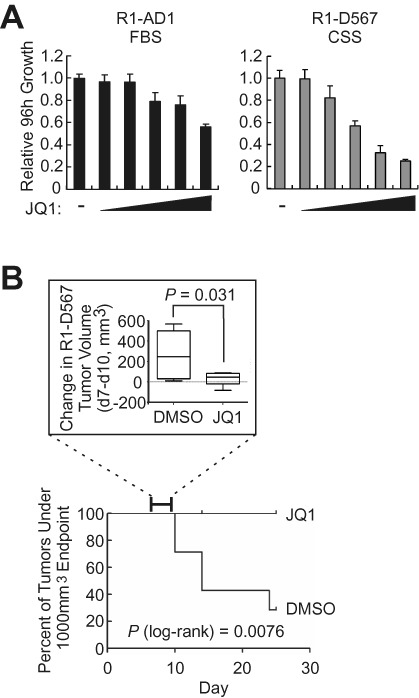
Inhibition of AR-V-driven PCa cell growth by JQ1. (**A**) Growth assays of R1-AD1 and R1-D567 cells in medium containing 10% FBS (whole serum) or CSS (steroid-depleted serum) with increasing doses of JQ1 (5 nM, 20 nM, 0.1 μM, 0.5 μM and 2 μM). (**B**) Subcutaneous R1-D567 xenograft tumors were established in intact male mice. When tumors reached 100 mm^3^, mice were randomized to treatment (JQ1, *n* = 7) or control (DMSO, *n* = 7) groups for 24 days. *Top*: change in tumor volume from day 7 to day 10 for DMSO versus JQ1 groups. Boxes represent first to third quartiles with median; whiskers represent range. *P* values were derived using a two-tailed *t*-test. *Bottom*: Kaplan–Meier analysis of DMSO versus JQ1 groups, representing time for tumors to reach 1000 mm^3^. *P* values were derived using a log-rank (Mantel–Cox) test.

We next tested whether this novel anti-AR activity of BET inhibition was associated with therapeutic efficacy within the context of AR-V-driven CRPC. JQ1 treatment inhibited androgen-dependent growth of R1-AD1 as well as constitutive growth of R1-D567 cells, but the effect on R1-D567 cells was more pronounced and occurred at lower concentrations (Figure 10A). Additionally, the growth rate of established R1-D567 xenograft tumors was reduced 1 week after initiating treatment of mice with JQ1, and continued treatment over 24 days extended the time to experimental endpoint (tumors reaching 1000 mm^3^) (Figure 10B). Collectively, these data provide preclinical evidence that therapeutic resistance driven by AR-Vs can be overcome with interventions that have selectivity in inhibiting AR-V expression and genome binding activity.

## DISCUSSION

AR-Vs are expressed widely in CRPC, and have been shown to drive resistance to AR-targeted therapies in diverse cell- and animal-based models (52). However, the mechanism of AR-V-based resistance has been a subject of debate, likely due to a lack of details regarding AR-V origin and function. In our previous work with heterogeneous CRPC tumors and cell lines, we showed that high-level AR-V expression, either concurrent with or exclusive from full-length AR, can often be ascribed to sub-clonal populations of tumor cells harboring underlying AR gene rearrangements (15–17). Additional studies have shown acute increases in AR-V mRNA and protein expression levels in response to castration and treatment with AR antagonists (43,53).

A recent study of tumor cells circulating in the blood of patients with CRPC found that mRNA expression of the AR-V7 splice variant was associated with resistance to abiraterone or enzalutamide (18). When quantitated, AR-V7 mRNA levels were consistently increased in circulating tumor cells collected post-treatment versus pre-treatment (18). Moreover, several patients whose circulating tumor cells were negative for AR-V7 expression converted to a positive AR-V7 expression profile during therapy (18). The R1-AD1/R1-D567 xenograft model of heterogeneous CRPC employed in our study displayed similar dynamic changes in AR-V expression during experimental therapy, indicating this model could provide disease-relevant insights to AR-V function in tumor progression.

To this end, the R1-AD1/R1-D567 model was used as an exceptionally clean model system for comparing functions of AR and a prototype AR-V, because both AR species are expressed independently but from the same endogenous AR locus. This model enabled the design of parallel ChIP-seq workflows utilizing the same antibody, and allowed us to avoid AR/AR-V knock-down or overexpression strategies. ChIP-seq with this R1-AD1/R1-D567 model revealed that ARv567es displayed a genome-wide binding preference for high-affinity AREs, which are the same sites preferentially bound by androgen-activated AR. Using *in vitro* binding assays, we found that ARv567es and AR-V7 engagement with these AREs was dimerization-dependent, requiring both ARE half-sites and D-box residues in the second zinc finger of the AR DBD, which is also analogous to androgen-activated AR (48,54)

It should be pointed out that a limitation of using the R1-AD1/R1-D567 model was the inability to address the role of potential interactions between AR and AR-Vs when they are co-expressed in the same cell, including heterodimerization on AREs. This will be important for future studies, especially given the discrepant reports in the literature on whether AR and AR-Vs heterodimerize when they are co-expressed (12,55). In this respect, the current study provides an important baseline, providing strong evidence for the notion that AR-Vs independently support therapeutic resistance in CRPC by recapitulating AR chromatin binding events that are otherwise lost or suppressed during endocrine therapy. This is also in agreement with microarray-based experiments, which have shown that AR-Vs can support constitutive expression of the broad androgen/AR transcriptional program (16,17).

Interestingly, an additional property that has been ascribed to AR-Vs is the regulation of a distinct subset of transcriptional targets that would be expected to provide a higher proliferative capacity and/or tumorigenic advantage to AR-V-expressing cells (36). Distinct AR-V transcriptional targets have been proposed to be the result of distinct AR-V chromatin binding sites (10,37,46,56). However, our genome-wide ChIP-seq analyses as well as direct interrogation of candidate loci (including UBE2C, a target that prior studies deemed to be unique to AR-Vs) did not provide support for the concept of distinct genomic AR-V binding sites. Nevertheless, our data do not completely rule out the existence of AR-V-specific gene targets for two key reasons. First, the levels of ARv567es protein expressed in steroid deplete R1-D567 cells are lower than the levels of AR protein expressed in steroid replete R1-AD1 cells, which biased ChIP-seq sensitivity in favor of AR binding events. Second, this bias was likely exacerbated by the lower affinity of AR-Vs relative to AR for high-affinity AREs, which could be due to loss of stabilizing interactions between the AR LBD and DBD (57). Despite these limitations, our data strongly suggest that any distinct genomic AR-V binding events, if they do indeed exist, are not among the predominant AR-V binding events in CRPC cells. Technical optimization will be required if these putative minor sites are to be discovered, defined, and characterized on a genome-wide basis.

The absence of a LBD presents a formidable challenge for direct inhibition of AR-Vs in CRPC. Our work has highlighted the low-affinity AR-V:ARE interaction as a potential vulnerability that could be exploited to interfere with constitutive AR-V function. JQ1, which inhibits the epigenetic reader function of BET family proteins, has increased potency in AR-driven PCa cell lines compared with AR-independent PCa cell lines (28,41). This was shown to be the result of direct interactions between BRD4 and the AR NH_2_-terminal domain, leading to genome-wide JQ1-mediated inhibition of AR binding to chromatin enhancers and impaired androgen induction of downstream targets (41). However, BET family proteins display only 10% overlap in binding with genomic AR binding sites (41), which appears to be at least partially inconsistent with the ability of JQ1 to block the broad androgen/AR transcriptional program. Our data revealing that BET inhibition down-regulates AR expression and thereby inhibits AR chromatin binding even at sites not associated with BET family proteins could potentially explain this discrepancy. In particular, this finding provides a logical mechanism for the observed dual actions of BET inhibitors repressing AR-activated genes and de-repressing AR-repressed genes. Mechanistically, we observed enrichment of H3K27Ac and BRD2, 3 and 4 near the transcription start site of the AR gene, which are chromatin features shared with other genes that display disproportionate sensitivity to JQ1 including MYC and CCND2 (50,51). Moreover, we found that treatment with JQ1 disrupted BRD2 binding to the AR 5′utr, which provides initial evidence for AR gene regulation by BRD2.

In conclusion, this study describes a therapeutic resistance mechanism in CRPC whereby AR-Vs constitutively engage and transcriptionally regulate canonical AREs as dimers. This study further provides pre-clinical proof-of-principle that challenges inherent with direct targeting of AR-Vs may be overcome by indirect targeting of AR-V expression and/or chromatin binding. Moreover, this study offers important insights into BET inhibitors in CRPC cells, most notably revealing down-regulation of AR and AR-V expression as a novel component of their anti-AR mechanism-of-action. This desirable activity is not achieved by any AR-targeted drugs currently in clinical use, which provides additional rationale for developing BET inhibitors as therapeutics for advanced PCa.

## ACCESSION NUMBERS

ChIP-seq data are available through NCBI's Gene Expression Omnibus (GSE61838).

## SUPPLEMENTARY DATA

Supplementary Data are available at NAR Online.

SUPPLEMENTARY DATA
