# Supplementary material for: Targeting chromatin binding regulation of constitutively active AR variants to overcome prostate cancer resistance to endocrine-based therapies
Source: Nucleic Acids Res. 2015 Apr 23;43(12):5880–97. doi: 10.1093/nar/gkv262 (PMC4499120; doi:10.1093/nar/gkv262)
Supplement: SUPPLEMENTARY DATA [file supp_43_12_5880_v2_index.html]

Targeting chromatin binding regulation of constitutively active AR variants to overcome prostate cancer resistance to endocrine-based therapies — Targeting chromatin binding regulation of constitutively active AR variants to overcome prostate cancer resistance to endocrine-based therapies — SUPPLEMENTARY DATA 

# Targeting chromatin binding regulation of constitutively active AR variants to overcome prostate cancer resistance to endocrine-based therapies

## SUPPLEMENTARY DATA

**Files in this Data Supplement:**

- SUPPLEMENTARY DATA
- SUPPLEMENTARY DATA
